# Models accounting for intention-behavior discordance in the physical activity domain: a user’s guide, content overview, and review of current evidence

**DOI:** 10.1186/s12966-015-0168-6

**Published:** 2015-02-07

**Authors:** Ryan E Rhodes, Christopher A Yao

**Affiliations:** Behavioural Medicine Laboratory, Faculty of Education, University of Victoria, PO Box 3015 STN CSC, Victoria, BC V8W 3P1 Canada

**Keywords:** Theory, Intention-behavior gap, Volition, Post-intention, Exercise, Action control

## Abstract

There is a growing concern among researchers with the limited effectiveness and yet subsequent stagnation of theories applied to physical activity (PA). One of the most highlighted areas of concern is the established gap between intention and PA, yet the considerable use of models that assume intention is the proximal antecedent of PA. The objective of this review was to: 1) provide a guide and thematic analysis of the available models that include constructs that address intention-behavior discordance and 2) highlight the evidence for these structures in the PA domain. A literature search was conducted among 13 major databases to locate relevant models and PA studies published before August 2014. Sixteen models were identified and nine overall themes for post-intentional constructs were created. Of the 16 models, eight were applied to 36 PA studies. Early evidence supported maintenance self-efficacy, behavioral regulation strategies, affective judgments, perceived control/opportunity, habit, and extraversion as reliable predictors of post-intention PA. Several intention-behavior discordance models exist within the literature, but are not used frequently. Further efforts are needed to test these models, preferably with experimental designs.

**Electronic supplementary material:**

The online version of this article (doi:10.1186/s12966-015-0168-6) contains supplementary material, which is available to authorized users.

One is hard-pressed to overstate the health benefits of regular moderate to vigorous intensity physical activity (PA). Benefits include a considerably reduced risk of most major chronic diseases such as heart disease, type 2 diabetes, several cancers and musculoskeletal disorders [1,2] as well as the prevention and rehabilitation of psychological disorders such as depression and anxiety [3,4]. Unfortunately, very few people in most developed nations engage in enough PA to reap a considerable effect on their health. For example, recent population-level assessments of PA using accelerometry suggest that over 80% of North Americans do not meet the guidelines for public health [5,6]. Clearly, PA promotion efforts are needed.

Theoretical understanding of the determinants behind PA has been a line of research inquiry for over 30 years [7]. The premise behind this research is that a sound understanding of PA determinants will aid in intervention success and theories represent an organizing framework to provide structure, function and common nomenclature to critical variables under study [8,9]. Many of the most researched theories applied to understanding PA include intention as the most proximal antecedent to PA. These include, but are not limited to, the theory of planned behavior [10], theory of reasoned action [11], protection motivation theory [12], social cognitive theory [13], variants of self-determination theory [14] and even stage models such as the transtheoretical model, where stage of change is an intention-behavior hybrid construct [15].

Tests of the intention construct and its relationship with PA have solid validation. For example, the most recent meta-analysis of the theory of planned behavior applied to PA showed *r* = .48 [16], which places this relationship within the medium-sized effect range [17]. Still, the finding also suggests that 77% of the variance in PA is unexplained. The relationship is also further attenuated when examining change in PA (i.e., controlling for past PA), which is arguably far more accurate when attempting to understand intention and its role in behavior change [8,18]; the relationship between PA and intention reduces to *r* = .22, which suggests a borderline meaningful effect [17].

Examinations of the absolute, rather than relative, value of intention-behavior relations have also shown considerable discordance. For example, experimental manipulations that increase PA intention (*d* = .45) result in much lower, and clinically less meaningful increases in PA (*d* = .15) [19]. Dichotomization of the intention and PA relationship around public health guidelines also showed that 48% of intenders failed to follow-through with PA [20]. Perhaps most important, is the lowered practical value of theories that place intention as the proximal antecedent of PA. It is extremely common for participants in PA interventions to report to the trial with high intentions at baseline (i.e., often the driving reason for study participation), yet low PA. This phenomenon poses a challenge to intention-based theories because those intentions are considered the proximal variable to behavioral enactment. Taken together, this line of research has prompted researchers to call intention-behavior discordance “the intention-behavior gap.”

The point here is not to doom intention-based theories or the intention construct. Indeed, research into intention-PA discordance clearly shows that intention is a necessary construct for behavioral enactment; almost no one reports performing PA with low intention [20]. Still, almost half of intenders do not follow-through with their intentions, which suggests that intention is a necessary, but insufficient construct for many when enacting behavior. An understanding of the translation of intentions into behavior therefore seems paramount to improve upon our theoretical frameworks in PA [21].

The separation of intention formation from intention translation, or what is sometimes referred to as action control [22], is not new. Indeed, conceptualizations of the distinctions between these concepts can be traced to the very early 20th century [23] and they were contemplated carefully in the mid-20th century [24], but models of intention formation have received the bulk of attention in the research community since that time. Contemporary research on the intention-behavior “gap,” however, has begun to shift some researchers and theorists back to an investigation of post-intentional constructs. In this relatively early phase of re-interest into intention-translation, it would seem helpful to collect the models available to PA researchers and practitioners that attempt to account for intention-behavior discordance. Comprehensive reviews of theories related to PA have mentioned some of these intention-translation models in passing commentary e.g., [25,26] but we are unaware of a collective review of these models. Thus, the purpose of this review was to 1) provide a user’s guide to the available models, frameworks, and theories^1^ that specifically include a post-intentional construct or constructs with a content analysis of the various constructs proposed to account for intention-behavior discordance and 2) highlight the available evidence for these structures when applied to the PA domain. The review is meant to be more descriptive than evaluative of these models at this phase and ultimately to raise awareness and empower PA researchers and practitioners with options for models that attempt to account for intention-behavior discordance.

## Method

### Eligibility criteria

Our literature search unfolded in a two-step process based on the purposes of the paper. First, we needed to identify what models offer a pre- and post-intentional conceptualization. To achieve this aim, an appropriate model for this review had to include the following criteria: First, the model had to be identified by a title or nomenclature to its identity. Thus, models tested as mere one-off tests of intention-based models (e.g., does personality mediate intention) were excluded. Second, the model had to include the intention construct within its frame in order to specifically demonstrate a post-intentional mechanism and intention needed to be a cognitive construct. Thus, models where intention formation may be implied, but not specifically articulated such as control theory [27] or dynamic action theory [28] were excluded. Further, models like the transtheoretical model [15], where the delineation between intent, plans and behavior are not clear (e.g., the preparation stage is marked by intent but also by some behavioral action and organizational/preparatory behaviors) were also excluded. Third, the model needed to have at least one construct where the central mechanisms were theorized as post-intentional. This could take three forms: 1) constructs proposed as mediators of intention-behavior relations, 2) constructs proposed as moderators of intention-behavior relations, and 3) constructs unrelated to intention in the theory that directly determine behavior. The latter consideration above was the aspect that needed the most careful scrutiny. Constructs in models that are not specifically stated as independent from intention formation did not meet our inclusion criteria, such as theory of planned behavior’s perceived behavioral control construct, where perceived behavioral control is hypothesized as an antecedent to intention and a potential direct determinant to behavior [10]. The second step in our review was to identify the application of all models found in step one to the PA domain. Eligible studies included the following: 1) the use of the identified intention-based model with post-intentional constructs and 2) a measurement of PA as a primary outcome. Studies were delimited in both searches to published works written (or translated) to English.

### Search strategy

A comprehensive literature search was conducted to identify potential models that integrated post-intentional constructs and have been previously applied to health behaviors. Health behaviors of interest included PA/exercise, healthy eating, smoking cessation, flossing, sunscreen use, vaccination, HIV prevention, or cancer screening. Major databases (Academic Search Complete, Academic Search Premier, AgeLine, CINAHL, Health Source, Medline, Alt HealthWatch, Health Technology Assessment, PsychINFO, PubMed, Social Sciences, SPORTDiscus and Web of Science) were used to search for any articles published prior to August 2014. A combination of the following key words that were used included: model, theory, framework, health behavior, physical activity, exercise, dieting, nutrition, healthy eating, smoking, flossing, cancer screening, HIV prevention, condom use, vaccination, immunization, volition, action, behavior, intention, post-intention, intention-behavior gap, mediator, and moderator. One author conducted the search, and reference lists of included studies were manually cross-referenced. Previously identified models from the authors’ personal records were added to the search. To enhance the comprehensiveness of our review, we also examined all 83 theories outlined in Michie and colleagues’ book, “ABC of Behavior Change Theories” [9]. This book has amalgamated many theories published to date and authored by experts in various fields.

The secondary search to identify the applications of the identified models in PA utilized the aforementioned databases. Keywords used in this search included the specific name of the model (e.g., health action process approach), exercise, and physical activity. All the reference lists of the studies included were carefully inspected to locate any additional studies.

### Data abstraction and analysis

Each author evaluated the identified models separately to determine its eligibility for the review. Once deemed eligible and a consensus was reached, all constructs relevant to intention-behavior discordance were themed. Our thematic analysis approach followed an iterative process. We first constructed a list and definition of the properties of prototypical constructs present in the most popular models in health and social behavior to use as a frame of reference [13,29,30]. Constructs from the identified models were then compared to this list and subsequently scored as one of these constructs or added to the list as an additional construct. Once an initial set of themes had been created, analysis shifted toward identifying broader patterns– that is, establishing the number of themes deemed sufficient to capture the essence of the main constructs in each model, followed by creating a label and description of each. Through this process, themes deemed sufficiently similar to one another were combined. All constructs were abstracted and categorized into themes independently and then compared to reach consensus.

In our subsequent analysis, the abstracted information included authors, country, sample (number, age, and gender), study design, model/framework/theory used, the use of the full model and post-intentional constructs, and any significant post-intentional predictors. Evidence was organized in observational and experimental categories and significant and meaningful associations by construct were tallied. Themes were created if three or more studies were present based on the prior review methodology by Rhodes and colleagues e.g., [31-33]. Based on Sallis et al.’s [34] rubric for determining an association among studies, a construct was considered to have a positive association (+) if greater than 59% of studies supported prediction of PA (or PA change); a negative association (−) if greater than 59% of studies supported a decrease in PA; inconclusive (?) if 34-59% of studies found any association, and no association (0) if less than 34% of studies showed any association. When analyzing the variables, both 1) statistical significance (*p* < .05) and a meaningful effect size [35,36] (*d* > .19; *r* > .09; *OR* > 1.49) needed to be present in order for a predictor to have either a positive or negative association with PA. Studies where effect size could not be determined were scored on significance value alone.

## Results

**Figure 1:**
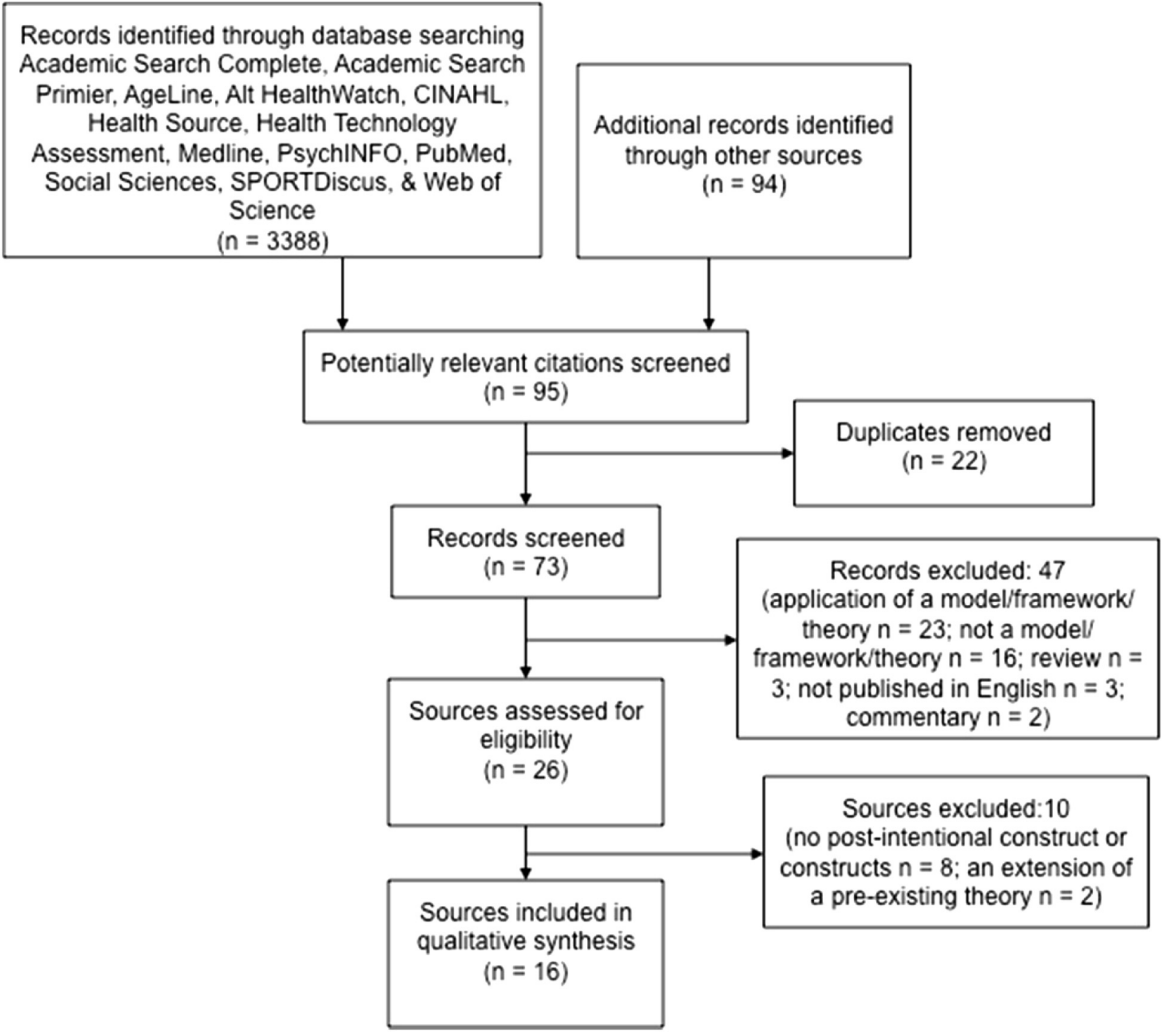
**Initial search for models, frameworks, and theories that address intention-behavior discordance.**

Details from the literature search for intention discordance models are described in a flow-diagram in Figure 1. The initial search yielded 3,388 potentially relevant records. Most papers were excluded because the study contained no indication of a specific model, or contained a model that did not address intention-behavior discordance. Twenty-six abstracts and full-text reports were obtained and reviewed. A total of 16 sources passed the eligibility criteria and were included in the initial part of the review.

All of these models were published in English between 1980 and 2014. The models originated from a range of countries, which included Germany, United States, Canada, Australia, Netherlands, Denmark and the United Kingdom. Most of the theoretical models, namely the ecological model for preventing type 2 diabetes in minority youth [37], health action process approach (HAPA) [38], precaution adoption process model [39], and temporal self-regulation theory [40], have simultaneously focused their initial efforts on a variety of health behaviors (e.g., diet and nutrition, PA, smoking cessation). Whereas, the integrated behavior change model [41], motivation-volition (MoVo) process model [42], and the action control framework, now referred to as the multi-process action control (M-PAC) framework [43], have been solely focused on PA behavior. Other models such as the integrated change (I-Change) model [44], information-motivation-behavioral skills model [45] and plans, responses, impulses, motives, and evaluations (PRIME) theory [46] have targeted health communication, HIV prevention and smoking cessation respectively. The action theory model of consumption [47] and motivation-ability-opportunity-behavior model [48] were originally conceptualized with consumerism as the targeted behavior. Finally, theories like the action control theory [22], Rubicon model of action phases [49], volitional model of goal-directed behaviors [50], and theory of interpersonal behavior [51] were generated as generic behavior models.

### Content analysis of intention-behavior discordance constructs

The two reviewers identified nine overall themes through content analysis of each theory (see Table 1). Each theme is detailed below:

**Table 1:** **Identified theories, model, and frameworks and intention-behavior discordance constructs (n = 16)**

**Theory**	**Volitional regulation beh.**	**Affect**	**Self-efficacy/beh. skills**	**Outcome expectancies**	**Selective attention**	**Endogenous factors**	**External factors**	**Habit**	**Identity**
Action Control Theory (Kuhl, 1984) [22]	√	√		√	√	√			
HAPA (Schwarzer, 2008) [38]	√		√						
Integrated Behavior-Change Model (Hager & Chatzisarantis, 2014) [41]	√								
I-Change Theory (de Vries et al., 2005) [44]	√		√				√		
Information-Motivation-Behavioral Skills Model (Fisher & Fisher, 1992) [45]	√		√						
Knowledge-Attitude-Behavior Model (Burnet et al., 2002) [37]			√						
Motivation-Ability-Opportunity-Behavior Model (Öllander & Thøgersen, 1995) [48]			√				√	√	
MoVo Process Model (Göhner et al., 2009) [42]	√				√				
M-PAC (Rhodes & de Bruijn, 2013) [20]	√	√	√			√	√	√	√
Precaution Adoption Process Model (Weinstein, 1988) [39]			√	√			√		
PRIME Theory (West, 2013) [46]	√	√		√		√	√	√	√
Rubicon Model of Action Phases (Heckhausen & Gollwitzer, 1987) [49]	√								
Theory of Consumption (Bagozzi, 2000) [47]	√		√					√	√
Theory of Interpersonal Behavior (Triandis, 1977) [51]						√	√	√	
Temporal Self-Regulation Theory (Hall & Fong, 2007) [40]						√	√	√	
Volitional Model of Goal-Directed Behavior (Bagozzi, 1992) [50]	√	√					√		√

Note. HAPA = Health Action Process Approach; M-PAC = Multi-Process Action Control Model.

#### Volitional regulation behaviors

Behaviors used to maintain or hone intentions featured prominently in 11 of the models identified [22,38,41-47,49,50]. These are described either broadly, with examples such as self-monitoring, scheduling, enlisting support, prioritizing and problem-solving around other behaviors as strategies an individual may use to maintain intentions [22,42,43,45,49,50] and/or specifically in terms of action plans or implementations intentions [38,41,44] and coping plans used to problem solve around difficulties in enacting the behavior [38].

#### Affect

Affect-based constructs were present in four of the reviewed models [22,43,46,50]. Affective judgments (e.g., enjoyment, pleasure) about the behavior itself and the motivational influence these have to maintain or abandon intentioned behavior were featured in action control theory [22], the volitional model of goal-directed behaviors [50], M-PAC [43], and PRIME theory [46]. Affect control in the form of shunting undesired general affective states in order to maintain one’s intentions featured in action control theory [22].

#### Self-efficacy/behavioral skills

Different types of self-efficacy variants are present in eight of the models reviewed [37-39,43-45,47,48]. The knowledge-attitude-behavior model [37], motivation-ability-opportunity-behavior model [48], and the precaution adoption process model [39] suggest that self-efficacy is a moderator of intention translation, with high self-efficacy needed to translate intentions into action. M-PAC suggests that control in the form of skills/ability, akin to task self-efficacy [52], impacts intention-translation with a threshold effect, where higher abilities may be needed to translate an intention into behavior than form an intention [43]. A similar construct of skills was proposed to mediate and moderate the intention and behavior relationship in the I-Change model [44] and mediate motivation-behavior relations in the information-motivation-behavioral skills model [45]. By contrast, the HAPA [38] suggests that two types of self-efficacy aid in translating intentions into behavior. Maintenance self-efficacy represents the confidence one can perform a behavior given various barriers. Recovery self-efficacy represents an individual’s confidence in performing the behavior under relapse or brief periods of non-performance. Finally, the theory of consumption positions perceived control as an antecedent of desire to achieve an intention, implementation intentions, volitional control strategies used in trying the behavior, and behavior itself [47].

#### Outcome expectations

Action control theory [22], PRIME theory [46], and precaution adoption process model [39] identified the use of outcome expectations in the role of translating intentions into behavior. These models suggest that conscious reminders of the expected outcomes from a behavioral act can be used to help bolster motivation.

#### Selective attention

Two of the models identify selective attention processes in maintaining intentions and following thorough with behavior [22,42]. This concept features prominently in action control theory [22] where selective processing of new information and competing intentions, and inhibition from over-processing information about the intended behavior are hypothesized as critical in order to translate intentions into behavior. Gӧhner et al.’s [42] MoVo process model also considers selective processing as crucial to moderating intention-behavior relations in the form of intention-shielding properties.

#### Endogenous factors

Stable individual differences featured in five of the models of intention-behavior discordance [22,40,43,46,51]. The theory of interpersonal behavior [51] considers individual arousal level as an independent system from intention that may facilitate or impede behavior. Action control theory [22] suggests that individuals have predispositions toward being either action-oriented and following through on intentions, or state oriented and maintaining the status quo. M-PAC [43] suggests that PA intention translation is affected by the personality traits of conscientiousness and extraversion, where those higher in these personality traits are more likely to follow-through with their intentions due to dispositional drive systems of achievement/order and energy-level respectively. Another unique consideration is Hall and Fong’s [40] construct of self-regulatory capacity in their temporal self-regulation theory, a biologically-based system of physiological energy and executive function capacity, that is proposed to moderate intentions and exert its own independent effect on behavior. Finally, West’s PRIME theory [46] suggest that dispositions are characteristics of the motivational system that govern its operation in stability and context sensitivity, thus affecting how intention gets translated into action.

#### External factors

Environmental and other facilitating/inhibiting conditions feature in eight of the models reviewed [39,40,43,44,46,48,50,51]. In Triandis’ [51] theory of interpersonal behavior and Baggozi’s [50] volitional model of goal-directed behaviors, facilitating (or inhibiting) environmental conditions are proposed to determine behavior as an independent system from intention. The M-PAC model suggests that opportunity to act (i.e., time and access) impacts intention-translation with a threshold effect, where greater opportunities to act are needed to translate an intention into behavior than form the original intention [43]. The precaution adoption process model [39] highlights that time, resources, competing opportunities all impact translation of intention into behavior. Finally, de Vries et al. [44] in their I-Change model, Öllander and Thørgersen [48] in their motivation-ability-opportunity-behavior model, West’s [46] PRIME theory, and Hall and Fong’s [40] temporal self-regulation theory suggest that external barriers (e.g., environmental and social) will moderate the intention-behavior relationship, with more barriers resulting in an attenuation of the intention-behavior link.

#### Habit

Six models suggest processes below conscious thinking are critical [40,43,47,48,51,53]. The theory of interpersonal behavior [51] proposes that habit, defined as automatic responses to cues from a patterned and learned behavior will determine behavior independent of intention. Bagozzi’s [47] model of consumption suggests that frequency and recency of behavior impact both the behavioral processes employed to enact a behavior and the behavior itself. The M-PAC [43] and motivation-ability-opportunity-behavior model [48] suggest that habit helps bind intended behavior to behavioral action. PRIME theory [46] highlights the importance of routine to the translation of plans (i.e., intentions) into action. Finally, Hall and Fong’s [40] temporal self-regulation theory proposes that behavioral pre-potency, a construct almost identical in conception to habit, will moderate the intention-behavior relationship and exert its own influence on behavior.

#### Identity

Four models suggest that role identity formation with a behavior is related to intention translation [43,47,50,53]. The M-PAC model [43] and Bagozzi’s volitional model of goal-directed behaviors [50] and theory of consumption [47] suggest that identity serves to preserve an intended behavior through the process of reducing affective/cognitive dissonance in contexts that trigger an awareness of one’s role identity (i.e. exerciser) with a discrepant action (e.g., not exercising). PRIME theory [46] contends that identity serves in a similar capacity for translating plans into action. Specifically, identity is thought to be the ultimate source of self-regulation.

#### Evaluation of the models of intention-behavior discordance applied to physical activity

**Figure 2:**
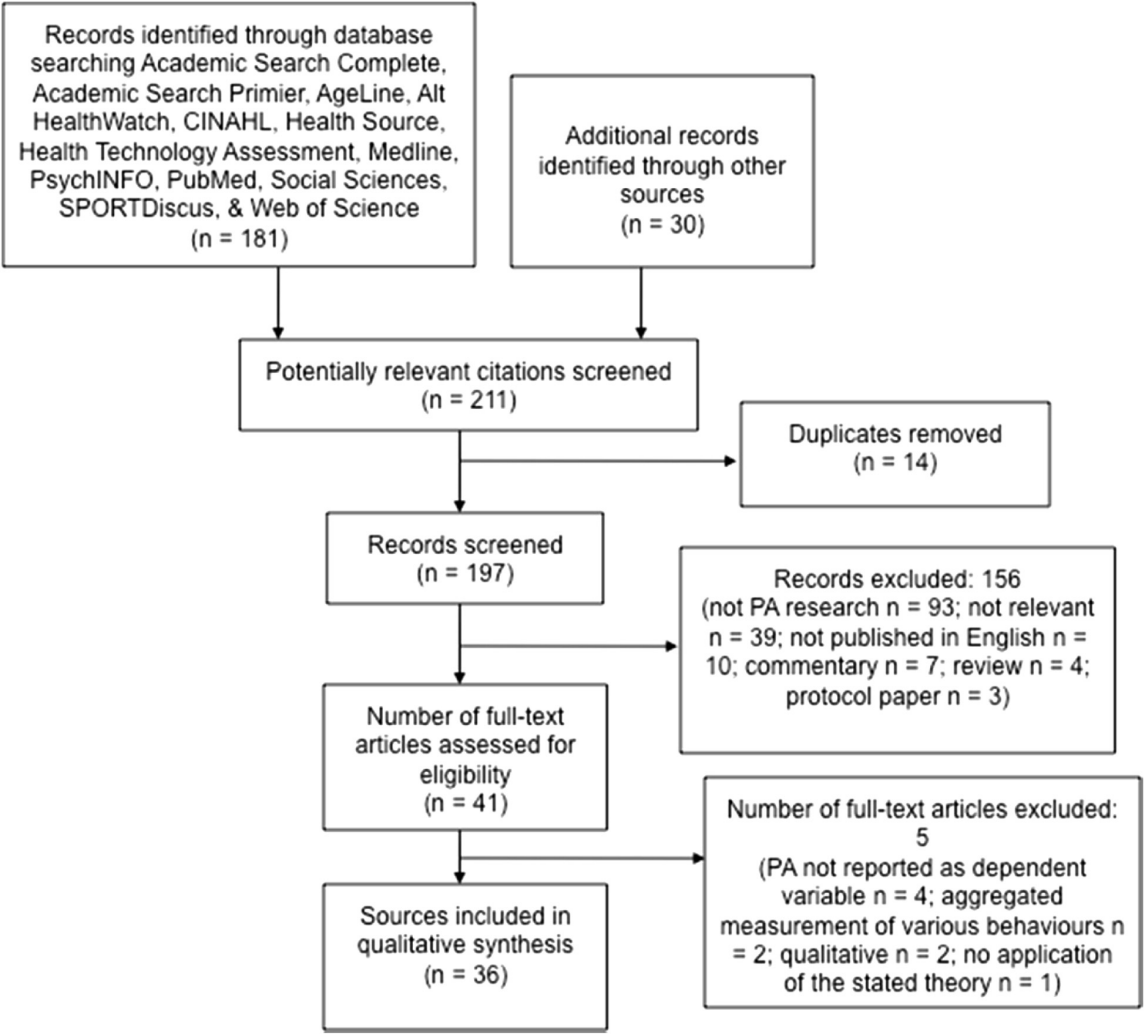
**Search for individual studies that utilize models, frameworks with post-intentional constructs.**

For the subsequent analysis, the literature search generated 181 relevant studies. Studies were predominantly excluded due to no concentration on PA behavior, no relevancy to intention-behavior discordance, or published in another language other than English. Overall, a total of 41 full-text articles were reviewed and assessed for eligibility. Of these, 36 studies were eligible for the analysis. Further details are outlined in Figure 2.

Only eight of the 16 previously identified theories were applied to PA (see Table 2 and Additional file 1: Table S1). Most commonly used, the HAPA model was applied to 12 observational studies and three intervention-based studies, while the M-PAC framework was used in 11 correlational studies and one experimental study. Less commonly applied theoretical models were the theory of interpersonal behavior [54], precaution adoption process model [55,56], I-Change model [57], information-motivation-behavioral skills model [58,59], MoVo process model [60,61], and temporal self-regulation theory [62]. Of these studies, five were observational and four were experimental. Due to the limited applications of these models, only the M-PAC model, and HAPA model will be discussed in further detail.

**Table 2:** **Descriptive summary of the individual studies included in the analysis (n = 36)**

**Model, framework, or theory**	**Total number of studies**	**Design**		**Use of all post-intentional constructs**
		Observational	Experimental	
Action Control Theory	0	-	-	-
HAPA	15	12	3	5/15
Integrated Behavior-Change Model	0	-	-	-
I-Change Theory	1	0	1	0/1
Information-Motivation-Behavioral Skills Model	2	1	1	1/2
Knowledge-Attitude-Behavior Model	0	-	-	-
Motivation-Ability-Opportunity-Behaviour Model	0	-	-	-
MoVo Process Model	2	0	2	1/2
M-PAC	12	11	1	0/12
Precaution Adoption Process Model	2	2	0	1/2
PRIME Theory	0	-	-	-
Rubicon Model of Action Phases	0	-	-	-
Theory of Consumption	0	-	-	-
Theory of Interpersonal Behavior	1	1	-	1/1
Temporal Self-Regulation Theory	1	1	0	0/1
Volitional Model of Goal-Directed Behavior	0	-	-	-

Note. HAPA = Health Action Process Approach; M-PAC = Multi-Process Action Control Model.

#### Health action process approach

Schwarzer’s [38] HAPA model was developed with specific aims to address intention-behavior discordance among health behaviors. The model differentiates between a pre-intentional phase of motivation and a post-intentional phase of volition for behavioral enactment. Action self-efficacy (perceived capability to perform the behavior), perception of risk from inaction, and outcome expectancies (expected consequences) are considered the determinants of intention, while coping-self-efficacy (confidence to handle barriers), action planning (detailed instructions of the behavioral enactment), coping planning (plans to handle barriers), and recovery self-efficacy (confidence to handle resume the behavior after set-backs) are considered the post-intentional determinants of behavior. The HAPA model also suggests a process of initiation to maintenance, where recovery self-efficacy is critical during behavioral maintenance.

Table 3 details the findings by HAPA constructs of the 12 studies, yet 14 independent samples that have formally applied the model to predict PA [63-74]. Three studies have tested the entire original model suggested by Schwarzer [38] in a single study i.e., [67,71,74], and enough studies are available to evaluate preliminary findings for the utility of each construct.

**Table 3:** **Summary of the post-intentional predictors in physical activity**

	**Predictor**	**# of studies**	**Association**
**Model/framework/theory – observational studies**			
HAPA	Maintenance SE-PA	8/12	+
*Two studies with two independent samples	Maintenance SE-Action Planning	2/6	0
	Maintenance SE-Coping Planning	1/2	n/a
	Maintenance SE-Planning	3/3	+
	Recovery SE-PA	2/6	0
	Action Planning-PA	2/8	0
	Coping Planning-PA	2/3	+
	Planning-PA	1/5	0
Information-Motivation-Behavioral Skills Model	Cognitive Behavioral Skills-PA	1//1	?
Precaution Adoption Process Model	Health Motivation-PA	1/1	n/a
	Knowledge-PA	1/1	n/a
M-PAC	Affective Attitude-PA	7/8	+
	Conscientiousness-PA	1/3	0
	Extraversion-PA	2/3	+
	Habit-PA	3/3	+
	Instrumental Attitude-PA	1/10	0
	Perceived Behavioral Control-PA	8/10	+
	Self-Regulation-PA	4/5	+
Theory of Interpersonal Behavior	Habit-PA	1/1	0
Temporal Self-Regulation Theory	-	n/a	n/a
**Model/framework/theory – experimental studies**			
HAPA	Planning-PA	2/3	+
I-Change Model	Action Planning-PA	0/1	n/a
Information-Motivation-Behavioral Skills Model	Behavioral Skills-PA	0/1	n/a
MoVo Process Model	Implementation Intentions-PA	2/2	n/a
	Volitional Shielding-PA	2/2	n/a
	Situational Cues-PA	1/2	n/a
M-PAC	Action Planning-PA	1/1	n/a

Note. At least three studies were required for a theme. + = positive association (>59% of studies), − = negative association (>59% of studies), ? = indeterminate (34-59% of studies showing an association) and 0 = no association (<34% of studies showing any association). PA = physical activity; n/a = not applicable; SE = self-efficacy.

Eight of nine studies have supported the predictive path of action self-efficacy on intention [63-70,74], most with large effect sizes (i.e., *β* > .50), demonstrating this as a reliable finding. Nine [63,64,67-69,71,73,74] and two [65,68] of 13 samples have shown support for the path of outcome expectations and risk perceptions on intention, respectively. The results suggest that outcome expectations may be useful in understanding PA intention, while risk perceptions may not.

Of the key constructs accounting for intention-behavior discordance, maintenance self-efficacy has had relatively strong support with eight [63,65,66,68,69,73,74] significant tests of 12 possible samples [64,67,71,72] (see Table 3). By contrast, recovery self-efficacy has shown a significant path in two [67,72] of six samples [70 samples 1 and 2, 71, 74]. Action planning was measured in eight samples [63,64,66,67,69,70,73], yet only two of these showed a significant path [66,73]. Only three samples have specifically applied coping planning and two supported a significant path to PA [70 samples 1 and 2] while the other was not significant or of meaningful magnitude (*β* < .10) [67]. Finally, of the five samples that employed an aggregate of coping and action planning [65,68,71,74], only one sample indicated this as a significant predictor of PA [71]. The results support maintenance self-efficacy as a post-intentional predictor of PA and suggest that coping planning may have utility, but action planning and an aggregate of action planning and coping planning does not appear to be useful to predict for intention-behavior discordance as proposed by the HAPA model.

Three intervention studies have applied the HAPA to promote PA [75-77]. All three have intervened upon the action and coping planning constructs and not the maintenance and recovery self-efficacy constructs. These studies have varied in participants from pregnant women [77] to obese patients [75] and a general population sample [76]. Two of the three studies have shown both statistically significant and meaningful (*d* > .20) increases in PA compared to control groups [76,77], yet only Gaston and Prapravessis [77] were able to demonstrate that the changes in PA were from changes in planning. The study by Ströbl and colleagues did not show meaningful effect-sized changes in PA, yet the difference in this study may be attributable to the six and 12 month post-intervention assessment compared to both Lippke et al. [76] and Gaston and Prapravessis [77], who examined planning effectiveness on PA over a one month period. Taken together, the results support HAPA’s potential effectiveness of action and coping planning, at least over a short duration, in order to increase PA.

#### Multi-process action control model

The M-PAC conceptual model proposed around the action control framework by Rhodes and de Bruijn [43] defines intention as a binary decisional choice variable and not the intention strength (and breadth of the motivational spectrum) definition taken in many intention-based theories [78]. Intention choice is formed by instrumental attitude (or outcome expectations), affective attitude (or experiential expectations) and perceived control constructs of ability (physical skill/movement without injury) and opportunity (time/access). Subsequent translation of intention choice into PA, called action control, is thought to be determined by regulatory behaviors (e.g., coping planning, enlisting support, self-monitoring), particularly during the initial adoption of the behavior. Continuation of the behavior is thought to include the addition of more reflexive means of action control via habit (responses to cues) and identity (responses due to role activation) development as one maintains a behavioral pattern. Furthermore, affective attitude and perceived control constructs are considered non-linear during the intention-formation to action control process, where much higher values are considered necessary for successful action control than mere intention formation. Personality traits of conscientiousness and extraversion are considered potential endogenous antecedents of action control, where those higher in these personality traits are more likely to follow-through with their intentions due to dispositional drive systems of achievement/order and energy-level respectively. Environmental and social context are considered subsumed (i.e., mediated) by affective attitude and perceived control constructs during the action control process which may facilitate or inhibit behavior.

Table 3 details the aggregate results for 11 prior prediction-based tests of the action control framework [79-89] and one experimental test [90]. No single study to date, however, has examined the entire conceptual model suggested by Rhodes and de Bruijn [43].

Tests have supported the nonlinear effects of affective attitude and perceived control in 7/8 and 8/10 tests respectively [see 43 for the full review]. Further, instrumental attitude failed to predict action control in nine of 10 samples, supporting its absence in the conceptual model after the initial formation of the intention. Self-regulatory constructs were assessed in five tests to evaluate action control. Four of the five studies showed significant prediction including the behavioral processes of change [86,87] (i.e., strategies such as rewards, self-monitoring, enlisting support, and creating stimulus control), regulation over other leisure behaviors [85] and coping planning [90] but not action planning [79]. Three studies employed a habit construct (i.e., enacting PA from external cues, starting PA without deliberation) to predict action control and found support for the construct [79,85,88]. Finally, three studies employed personality trait measures of extraversion (i.e., sociability, positive affect, assertiveness, preference for lively activity) and conscientiousness (i.e., industriousness, orderliness, self-discipline) to predict action control framework and found two in support of extraversion [84,89] but only one test in support of conscientiousness [80]. Results examining the identity construct and the difference among constructs between adoption and maintenance have not been evaluated sufficiently to produce an outcome theme at present.

## Discussion

There is a growing concern among theoretical researchers with the limited effectiveness and yet subsequent stagnation of theories applied to health behaviors like PA [21,91-95]. At the top of this list are theories that position intention as the proximal predictor of behavior due to the well-established intention-behavior gap [19,20,78,96]. In the spirit of trying to move forward in theory development and application, the purpose of this review was to 1) provide a user’s guide and thematic analysis to the available models that specifically include a construct to explain intention-behavior discordance and 2) highlight the available evidence for these structures when applied to the PA domain.

Our review yielded 16 models that have positioned constructs in pre-intentional and post-intentional positions. When taken into context of the gamut of possible health behavior theories [9], this represents approximately 19% of known theoretical frameworks. Thus, a sizeable proportion of models are available for researchers attempting to account for the intention-behavior gap. Many of these models are relatively new – eight have been proposed within the last 10 years. Nevertheless, some have been available since the 1980s [22,39,49,51] or early 1990s [45,48,50]. Researchers have had the opportunity to test these theories in the PA domain for quite some time.

Despite 16 different models for researchers to choose from, our content analysis of constructs suggested considerable overlap in the kinds of factors that are being proposed to account for intention-behavior discordance and some redundancies with general intention models. The hallmark of many post-intentional models is the inclusion of volitional regulatory behaviors used to maintain or hone intentions. Theorists suggest that people who form intentions, need to then become strategic in order to implement their intentions across the backdrop of competing forces for their attention, motivation, and time. Eleven of these models included these behavioral regulation constructs, yet they differed from very specific concepts such as action plans or implementations intentions to a more general array of behaviors such as self-monitoring, scheduling, enlisting support, prioritizing and problem-solving. Volitional behaviors used to maintain or hone intentions have sound empirical evidence in the PA domain generally [97-101] and within a stage-based model such as the transtheoretical model of behavior change [102]. They also have high face validity, as participants who report for PA intervention trials often already have high intentions and seek strategic advice for the translation of these intentions into behavior [43].

Two post-intentional elements that were suggested in nearly half of the models included external conditions and/or endogenous factors. While these are at opposite ends of the spectrum in terms of individual vs. environment, they both share more immutable qualities that may either facilitate or thwart intention translation. For example, external conditions represent the opportunities an individual may have to enact PA as represented by the social and physical environment. This concept takes social ecology into consideration within post-intentional models, where its omission in several intention-based models has often been a criticism [103]. The concept also has support in the PA domain outside of these models, where a factor such as proximity to recreation facilities has been shown to moderate intention-behavior relations [104-106]. Endogenous factors represent individual differences in organizational/executive function, cravings and energy/arousal that may thwart or facilitate intention translation or enactment of behavior. Endogenous factors as constructs used to explain health behavior are not unique to post-intentional models, but the operation of these constructs take neuroscience, broadly construed, into consideration as a factor explaining the intention-behavior gap. Disposition has been shown to moderate the PA intention-behavior gap in several tests outside of these theories see [107] for a review, but application tests of several of the constructs proposed in these theories are needed.

More reflexive constructs such as habit (responses to cues) and identity (responses from role activation) were featured in about a third of the models. These are interesting concepts, not traditionally employed in many popular intention theories. These constructs suggest that intention translation may be tied to less reasoned or pre-meditated processes [108]. Both have had some evidence in the PA domain outside of these post-intentional models [109,110] but require sustained research attention.

Finally, specific motivational concepts such as specific types of self-efficacy, outcome expectations and affective judgments were also identified in many of the models. These do not represent particularly new concepts from prior intention-based models but the implication for these types of constructs is that intention translation requires either different forms of motivation than intention formation or a higher threshold of these motivational constructs. The presence/absence of these factors underscored some of the differences among these models in terms of how intention was operationalized. For example, some theories considered intention as the definitive motivational variable (intention strength), akin to the definition put forth by Fishbein and Ajzen [11]; thus, post-intentional constructs needed to represent concepts outside the motivational domain e.g., [40,42,49]. By contrast, other theorists positioned intention more within the dictionary meaning of an aim toward a behavior (intention choice) [111], thus allowing for motivational variables to affect that aim during intention translation [22,43,46,47,50]. Applied researchers will need to carefully consider how they view intention in order to select a theoretical frame.

The definition of behavior, the application of the theory for intervention, and the specific mechanisms accounting for intention-behavior discordance were also somewhat different across the various models. For example, HAPA [38], temporal self-regulation theory [40], and M-PAC [43] give some explicit consideration of adoption vs. maintenance behavior and how different constructs may affect behavior across time. Temporal self-regulation theory [40] appears to be an explanative model of behavior first and foremost, while information-motivation-behavioral skills model [45] is a simplified model for interventionists. Most of the other models were positioned somewhere in between these approaches. Finally, some models position their post-intention constructs as moderators of intention-behavior relations e.g., [37,41,48], others position them as direct mediators e.g., [38], while some position these constructs as separate systems impacting behavior independent of intention e.g.,[51]. Indeed, our content analysis suggested that Temporal self-regulation theory [40] and the theory of interpersonal behavior [51] contained relatively similar post-intentional constructs overall, but their proposed mechanisms were markedly different. Thus, applied researchers need to give attention to the specific suggestions of how to model each theory.

Our second purpose of the paper was to review and highlight the available evidence for these 16 models when applied to the PA domain. Perhaps the most important finding from this second purpose was a demonstration of how little these models have been applied to understand PA. Our application of theory in PA tends to extend to only a handful of approaches [112], none of which are among these models. We had anticipated this outcome, and it formed one of the central reasons for writing the paper as a user’s guide.

Thirty-six studies were identified for the 16 models, but 27 of these were from two of the models (HAPA and M-PAC). Eleven of the models had either one or zero applications in the PA domain making any kind of assessment too preliminary. There was enough research available to evaluate the early evidence for both M-PAC [43] and HAPA [38] in the PA domain. M-PAC is more of a methodological framework than a theory at this juncture and the results herein were actually used to create the proposed conceptual model post-hoc, as opposed to validating the model. Future tests and the use of its complete set of constructs are definitely warranted. Thus far, there is evidence that affective judgments, perceived control/opportunity, habit, and extraversion can reliably predict PA intention translation but there is not enough evidence to evaluate the adoption to maintenance transition proposed in the conceptual model or the role of identity. There is also not enough experimental work with the model to warrant an appraisal. Unlike M-PAC, HAPA has had much better independent assessment outside of the original studies used to create the model, but application of the full model was also limited. Interestingly, nearly half of the samples employing HAPA included clinical populations, suggesting good heterogeneity of its application although no marked difference in findings was present between clinical and nonclinical samples at this time. Of the key constructs accounting for intention-behavior discordance, maintenance self-efficacy has had relatively strong support as a predictor PA independent of intention. This suggests that how people cope with PA barriers may be essential to successful behavioral enactment. Action planning, on the other hand, has shown generally weak to negligible effects. Coping planning has fared better as a mediator of intention than action planning but its application has been limited. Experimental work with the model has also supported HAPA’s planning constructs (at least action planning and coping planning in tandem) for short-term PA change, but no studies were present to evaluate the maintenance and recovery self-efficacy constructs.

Taken together, intention-based theories have dominated the PA research landscape, and there has been a sluggishness to embrace models that attempt to account for intention-behavior discordance. Clearly, there are several models that attempt to account for this discordance within the literature, but they have not been used and validated. The reasons for the dominance of a few select theories in PA research may be from tradition (researchers trained from a similar set of supervisors), convenience (easier to test and publish with models of high use), lack of awareness, or general lack of innovation. Whatever the case, the models identified in this review propose several variables such as volitional strategies, social and environmental conditions, disposition, habit, identity, as well as affect, PA skill and selective attention processes that are not as well-defined in our traditional intention theories. Whether these models will serve to improve our interventions and/or explain PA better than the current state of research is unknown, yet early work with HAPA has shown some promise. At a minimum, many of these models may serve to better integrate extant interventions that already focus on volitional strategies, environmental change, social processes/dynamics and individual differences, etc. [100]. The results of this paper suggest there are certainly models that warrant testing, preferably with experimental designs, in the future.

Footnote 1: To simplify, we refer to models, theories, approaches or frameworks as “models” generically from this point forward.

## Additional file

Additional file 1: Table S1. Characteristics of physical activity studies that utilized models/frameworks/theories (n = 36).
