# Supplementary material for: Models accounting for intention-behavior discordance in the physical activity domain: a user’s guide, content overview, and review of current evidence
Source: Int J Behav Nutr Phys Act. 2015 Feb 7;12:9. doi: 10.1186/s12966-015-0168-6 (PMC4328062; doi:10.1186/s12966-015-0168-6)
Supplement: Additional file 1: Table S1. — Characteristics of physical activity studies that utilized models/frameworks/theories (n = 36). [file 12966_2015_168_MOESM1_ESM.docx]

Supplementary Table

*Characteristics of physical activity studies that utilized models/frameworks/theories (n = 36)*

| Author, year (country) | Sample Characteristics (# of participants, age, gender, study design) | Model, Framework or Theory Used | Predictor  Variables | Full Model Used | Use of All Post-intentional Constructs | Significant Post-intentional Predictors |
| --- | --- | --- | --- | --- | --- | --- |
| Barg et al., 2012  (United States) | N = 175  M_age_ = 52.0  All female  OBS | HAPA | Risk Perception  Outcome Expectancies  Preaction SE  Intention  Planning (action)  Maintenance SE | No | No | Maintenance SE-Action Planning .73, p < .01  Maintenance SE-PA .47, p < .01 |
| Blalock et al., 1996 (United States) | N = 452  All female  M_age_ = 39.7 yrs  OBS | PAP | Health Motivation  Perceived Severity  Barriers  Perceived Inconvenience  Exercise Health Concerns  Perceived Benefits  Subjective Norm  Osteoporosis Concern  Perceived Susceptibility  SE  Precaution Effectiveness  Osteoporosis Knowledge  Information Seeking | No | No | *none |
| Berli et al., 2013 (Switzerland) | N = 430  199 f, 231 m  M_age_ = 14.6 yrs  OBS | HAPA | Compensatory Health Beliefs  Risk Perception  Outcome Expectancies  SE (maintenance)  Intentions  Action Planning | No | No | Maintenance SE not significant predictor PA  Action Planning not significant predictor of PA |
| Caudroit et al., 2011 (France) | N = 120  79 f, 41 m  M_age_ = 49.0 yrs  OBS | HAPA | Risk Perception  Preaction SE  Outcome Expectancies  Intention  Planning (action, coping)  Maintenance SE | No | No | Maintenance SE-Planning .58, p < .001  Maintenance SE-PA .33, p < .001 |
| Chiu et al., 2011 (United States) | N = 195  170 f, 25 m  M_age_ = 47.4 yrs  OBS | HAPA | Severity  Action SE  Maintenance SE  Recovery SE  Outcome Expectancy  Risk Perception  Perceived Barriers  Planning (action, coping)  Intention | Yes | Yes | Planning-PA .38, p < .05  Maintenance SE-Planning .64, p < .05  Maintenance SE-PA not sig. predictor  Recovery SE-PA not sig. predictor |
| de Vries et al., 2008 (Netherlands) | N = 2827  1555 f, 1272 m  M_age_ = 49.0 yrs  INT | I-Change | Stages of Change  Action Plans  SE  Social Influence  Attitude | No | No | *explored the application of action planning to tailored messages  *action planning did not enhance the effects to the experimental conditions |
| Duan et al., 2011 (China) | N = 91  38 f, 53 m  M_age_ = 19.1 yrs  OBS | HAPA | Risk Perception  Outcome Expectations  SE  Intention | No | No | *the HAPA was compared to TTM for assessing PA stages  *HAPA was a more parsimonious stage approach |
| Elliott et al., 2007 (United States) | N = 94  70 f, 24 m  M_age_ = 40.0 yrs  OBS | PAP | Osteoporosis Knowledge  Health Beliefs  Risk Perception  SE | No | No | Exercise Knowledge-Adoption Stage F(2, 70) = .853  Health Motivation-Adoption Stage F(2, 70) = .878 |
| Fuchs et al., 2012 (Germany) | N = 220  125 f, 95 m  M_age_ = 51.1 yrs  INT | MoVo | SE  Outcome Expectations  Strength of Goal Intention  Self-concordance  Implementations  Volitional Intention Shielding | No | No | Adoption Model  Action Planning-PA .21, p < .01  Maintenance Model  Barrier Management-PA .23, p < .01 |
| Gaston & Prapavessis, 2012 | N = 60  All female  M_age_ = 51.1 yrs  INT | HAPA | Perceived Vulnerability  Perceived Severity  Response Efficacy  SE  Intention  Action Planning  Coping Planning | No | No | Action Planning and Planning (action + coping) groups significantly increased PA compared to the control group (p < .01) |
| Göhner et al., 2012 (Germany) | N = 315  244 f, 71 m  M_age_ = 50.6 yrs  INT | MoVo | SE  Strength of Goal Intention  Implementation Intentions | No | Yes | Implementation Intentions  Volitional Intention Shielding  Situational Cues |
| Kassavou et al., 2014 (United Kingdom) | N = 114  88 f, 26 m  20-89 yrs  OBS | HAPA | Recovery SE  Maintenance SE | No | No | Recovery SE-PA .21, p < .05  Maintenance SE not a sig. predictor of PA |
| Kelly et al., 2012 (United States) | N = 404  212 f, 192 m  M_age_ = 15.1 yrs  OBS | IMB | Community Factors  Intrapersonal Factors  Knowledge  Intentions  SE  Barriers  Cognitive-Behavioural Skills | Yes | Yes | Behavioural Skills-PA .31, p < .001 |
| Li, 2013 (Hong Kong) | N = 300  240 f, 60 m  M_age_ = 76.0 yrs  OBS | TST | Multidimensional Outcome Expectancy  Perceived Outcome Immediacy  Future-Time Perception  Self-Construal | No | No | People with high-immediate expectancy were more physically active |
| Lippke et al., 2010 (Germany) | N = 226  186 f, 40 m  M_age_ = 37.2 yrs  INT | HAPA | Risk Awareness  Outcome Expectancies  Intentions  Volitional SE (maintenance and recovery SE)  Planning (action) | No | No | For Intenders, action planning mediated behaviour change (p < .01) |
| Lippke & Plotnikoff, 2014 (Canada) | N = 1193  568 f, 625 m  M_age_ = 63.7 yrs  OBS | HAPA | Outcome Expectancies  Risk Perception  Action Planning  Maintenance SE | No | No | Action Planning-PA .10, p < .05  Maintenance SE-Action Planning .11, p < .05  Maintenance SE-PA .22, p < .05 |
| Osborn et al., 2010 (United States) | N = 91  68 f, 23 m  M_age_ = 57.7 yrs  INT | IMB | None | No | No | Behavioral Skills not a sig. predictor of PA |
| Parschau et al., 2014 (Germany) | N = 484  328 f, 156 m  M_age_ = 42.3 yrs  OBS | HAPA | Action Planning  Coping Planning  Maintenance SE  Motivational SE  Recovery SE  Positive Outcome Expectancies  Risk Perception  Intention | Yes | Yes | Maintenance SE-Coping Planning .31, p < .05  Recovery SE-PA .19, p < .05 |
| Perrier et al., 2012 (Canada) | N = 201  119 f, 82 m  M_age_ = 40.0 yrs  OBS | HAPA | Outcome Expectancy  Risk Perception  SE (preaction, maintenance, recovery)  Planning (action, coping) | Yes | Yes | Maintenance SE-PA .48, p < .01  Maintenance SE-Planning .57, p < .001  Planning not sig. predictor of PA  Recovery SE not sig. predictor of PA |
| Renner et al., 2007 (South Korea) | N = 697  358 f, 315 m  M_age_ = 32.0 yrs  OBS | HAPA | Risk Perception  Outcome Expectancies  Preaction SE  Maintenance SE  Intention  Action Planning  Coping Planning | No | No | Older Adults  Preaction SE-Planning .56, p < .01  Maintenance SE-PA .37, p < .01  Younger Adults  Preaction SE-PA .20, p < .05  Maintenance SE-PA .22, p < .01 |
| Rhodes et al., 2010 (Canada) | N = 85  Families  INT | M-PAC | Perceived Behavioural Control  Intention | No | No | Action Planning-PA |
| Rhodes et al., 2012 (Canada) | N = 263  183 f, 80 m  M_age_ = 24.0 yrs  OBS | M-PAC | Instrumental Attitude  Affective Attitude  Perceived Control  Cross-Behavioural Regulation  Intention | No | No | *only examined predictors of Action Control: Affective Attitude p < .01; Perceived Behavioural Control p < .01; Automaticity p < .01; Cross-behavioural regulation p < .01 |
| Scholz et al, 2009 (Germany) | N = 265  195 f, 70 m  M_age_ = 21.3 yrs  OBS | HAPA | Risk Awareness  Positive Outcome Expectancies  SE (maintenance)  Intentions  Action Planning  Action Control | No | No | Maintenance SE-PA .15, p < .05 |
| Ströbl et al., 2013 (Germany) | N = 341  153 f, 188 m  M_age_ = 40.0 yrs  INT | HAPA | Outcome Expectancy  Risk Perception  Intention  SE (preaction, maintenance, recovery)  Planning (coping, action) | Yes | Yes | Planning-PA .14, p < .001 |
| Valois et al., 1988 (Canada) | N = 166  66 f, 100 m  M_age_ = 39.7 yrs  OBS | TIB | Affect  Social Norm & Role  Personal Normative Belief  Facilitating Conditions  Habit  Intention | Yes | Yes | Habit-PA .31, p < .001 |
| Ziegelmann et al., 2007 (Germany) | N = 368  228 f, 140 m  M_age_ = 47.4 yrs  OBS | HAPA | Risk Perception  Preaction SE  Recovery SE  Positive Outcome Expectancies  Intention  Action Planning  Coping Planning | No | Yes | Young Adults  Coping Planning-PA .51, p < .001  Action Planning-PA .52, p < .001  Older Adults  Coping Planning-PA.52, p < .001  Action Planning-PA .48, p < .001 |

INT = Intervention; HAPA = Health Action Process Approach; MoVo = Motivation Volition Process Model; M-PAC = Multi-Process Action Control Model; OBS = Observational Study; PA = Physical Activity; PAP = Precaution Adoption Process Model; SE = Self-Efficacy; TIB = Theory of Interpersonal Behavior; TST = Temporal Self-Regulation Theory
